# Spinal AMPA receptors: Amenable players in central sensitization for chronic pain therapy?

**DOI:** 10.1080/19336950.2021.1885836

**Published:** 2021-02-10

**Authors:** Olga Kopach, Nana Voitenko

**Affiliations:** a Department of Sensory Signalling, Bogomoletz Institute of Physiology, Kyiv, Ukraine; b Kyiv Academic University, Kyiv, Ukraine; c Present Address: Department of Clinical and Experimental Epilepsy, Queen Square Institute of Neurology, University College London, London, UK

**Keywords:** AMPA receptors, AMPAR trafficking, GluA1-4 subunits, the dorsal horn (DH) of the spinal cord, sensory DH neurons, nociceptive circuits, chronic pain

## Abstract

The activity-dependent trafficking of AMPA receptors (AMPAR) mediates synaptic strength and plasticity, while the perturbed trafficking of the receptors of different subunit compositions has been linked to memory impairment and to causing neuropathology. In the spinal cord, nociceptive-induced changes in AMPAR trafficking determine the central sensitization of the dorsal horn (DH): changes in AMPAR subunit composition compromise the balance between synaptic excitation and inhibition, rendering interneurons hyperexcitable to afferent inputs, and promoting Ca^2+^ influx into the DH neurons, thereby amplifying neuronal hyperexcitability. The DH circuits become over-excitable and carry out aberrant sensory processing; this causes an increase in pain sensation in central sensory pathways, giving rise to chronic pain syndrome. Current knowledge of the contribution of spinal AMPAR to the cellular mechanisms relating to chronic pain provides opportunities for developing target-based therapies for chronic pain intervention.

## Introduction: Implication of spinal AMPAR for chronic pain

Chronic pain is a major healthcare issue worldwide; it has a serious impact on an individual’s life and work activities, as well as on society [1]. It has been estimated that about 20% of the population in Europe suffers from persistent or chronic pain [2,3], with even more in the United States, with a figure ranging from 20% to 40% [4]. This problem is growing each year, in both absolute numbers and in terms of its distribution across the global population [5]. The International Association for the Study of Pain (IASP) has announced the 2020 Global Year for the Prevention of Pain to help focus on disseminating pain prevention strategies among researchers, clinicians, and every involved party for a global view on pain. Through the cooperation between the IASP and the World Health Organization, the definition of chronic pain has been revised and will appear in the upcoming edition of the International Classification of Diseases in the form of a new classification: chronic pain as a disease in its own right [6,7]. In such a context, chronic pain requires specialized, targeted treatment, i.e. with no regard to the trigger(s) that give rise to the development of chronic pain syndrome. To date, however, there is no available therapy for curing chronic pain that is both effective and free of adverse effects.

After decades of studying chronic pain, it has emerged that mechanisms contributing to the development of chronic pain and its maintenance are multifaceted and complex; such mechanisms can involve versatile and intricate signaling pathways engaged in sensory processing, both in the periphery and in the central nervous system (CNS), as has been outlined in a number of exceptional reviews [8–10]. A plethora of alterations have been described in signaling cascades, synaptic and neuronal activity, nerve cell function and network integration – from individual proteins to ion channels and receptors – suggesting that the multiple discovered mechanisms can significantly contribute to perturbed sensory processing, resulting in pathological pain.

Among those, a class of glutamatergic receptors – the α-amino-3-hydroxy-5-methyl-4-isoxazolepropionic acid receptors (AMPAR) – is of great interest. This class of ionotropic receptors plays a critical role in synaptic strength and plasticity [11–13] and is considered to underpin learning and memory formation in the brain. In the spinal cord, these receptors mainly determine the central sensitization of the dorsal horn (DH) – a phenomenon which has been thought to represent a specific form of spinal plasticity and which underlies pain states of various origins [14–19] (for a review see [20,21]).

The implications of the role of spinal AMPAR in cellular mechanisms mediating the development and maintenance of chronic pain have emerged from several lines of experimental evidence. Studies from *in vivo* models of pain have clearly demonstrated that the pharmacological inhibition of spinal AMPAR significantly alleviates nociceptive hypersensitivity in animals experiencing pain of different origin: neuropathic [18], inflammatory [22–24], injury-induced [25,26] or postoperative pain [27–29]. Spinal AMPAR mediates sensory signaling, both at nociceptive and non-nociceptive circuits, and are heavily involved in the development [23,30,31] and maintenance of chronic pain [14,23,31–33]. Another line of evidence implicating AMPAR in terms of chronic pain originates from genetically engineered mouse lines, in which genetic deletion of a subunit alone (one of the four subunits, GluA1-4, which assemble into a tetramer of various combinations to make up the functional cation-permeable channel) clearly changed pain sensation in knock-out (KO) animals [32,34,35].

## Spinal AMPAR: Are they any different from those across the brain?

The key role that AMPAR plays in synaptic transmission and plasticity makes it difficult to use ablation of this class of receptors in order to manage chronic pain because it can cause severe dysfunctions in the CNS. In a wider context, studies using generalized genetic manipulations offer a basic picture of the specific roles of all four AMPAR subunits (GluA1-4). Using gene targeting to harbor the expression of the AMPAR subunits, it has been found that mice deficient in GluA2 exhibit impaired motor coordination and behavioral abnormalities [36] and died of seizures during the first weeks postnatal [37]. Interestingly, mice with the genetic deletion of GluA2 were able to express long-term potentiation (LTP) [36,38] – an experimental model of synaptic plasticity for learning and memory formation. Likewise, double KO animals lacking GluA2/A3 expressed LTP, but displayed a deficit in basal transmission [39]. Conversely, the genetic deletion of GluA1 resulted in impaired LTP [40] and robust short-term memory deficit [41–43], pointing to a central role of the GluA1 subunit in synaptic plasticity and LTP.

In the spinal cord pain pathways, LTP at the C-fiber synapses was documented following a high-frequency stimulation of primary afferents [44,45], a mode of presynaptic stimulation similar to that used for the hippocampal LTP induction [46,47], or in response to noxious peripheral stimuli [48,49]. Such LTP caused hyperalgesia that could last for several days [50]. Although it highlights the causality between LTP at the C-fiber synapses and chronic pain, whether and to what degree the GluA1-containing AMPAR are engaged in nociceptive-induced potentiation remains open to question.

A common stoichiometry of AMPAR – identified in the forebrain neurons – is the receptor composition of the two GluA1 and two GluA2 subunits [51]. The hetero-tetrameric composition has been confirmed using a single-cell genetic approach coupled with electrophysiology, and it was found that most receptors in the hippocampal pyramidal neurons are GluA1/A2 heteromers (~80%), with minor GluA2/A3 ones [52,53]. The GluA4 subunit is amply expressed by interneurons and is not involved in the fast transmission in the “mature” excitatory neurons of adults [54,55]. GluA2 homomers are not thought to occur naturally, and only a few GluA1 homomers (approximately 8%) were identified [39]. Conversely, most of the knowledge, if not all, with regard to the AMPAR subunit composition in the spinal cord has emerged from immunostaining with quantification analysis of the immunoreactivity of GluA1-4.

According to the immunofluorescence regional analysis, all four AMPAR subunits are expressed in the spinal cord [56,57]. Their distribution, however, is not uniform: a higher immunoreactivity of both GluA1 and GluA2 was found in the DH, showing the highest GluA1 density in the superficial DH (laminae I and II) – an area where most C-and Aδ-nociceptive afferents terminate to form synapses with the laminae I–II DH neurons for nociceptive information integration and processing (for a review see [8,58]) – while the GluA2 immunoreactivity is equally distributed throughout the DH [59–61]. In contrast, GluA3 was detected in deeper laminae (III to V), an area of the low-threshold afferent projections. Consequently, it can be interpreted that AMPAR in the DH predominantly consist of GluA1/A2 heteromers. Intriguingly, the GluA4 immunostaining was noticed in the lamina I NK1 receptor-positive projection neurons [60], a small group of large neurons which convey chronic nociceptive transmission from the periphery [62]. Studies identifying the GluA1-4 stoichiometry in different neuronal types, especially in nociceptive *vs*. non-nociceptive circuits, will be of great importance.

Despite the fact that the AMPAR subunit composition in the DH remains largely elusive, electron microscopy studies, combined with immunofluorescence, have provided insights into the subcellular distribution of GluA1/A2. Both GluA1 and GluA2 have been identified in the postsynaptic and extrasynaptic membranes of the superficial DH neurons [34,57,63]. At synapses, the receptors are concentrated in varied densities; their number depends on spine geometry, and ranges from tens to hundreds, and correlates with the synaptic strength [64,65]. For instance, an individual “mature” (mushroom-shaped) spine of a CA1 pyramidal neuron can host up to 150 AMPAR [66]. Likewise, the number of AMPAR in the extrasynaptic plasma membranes varies greatly. The density of extrasynaptic AMPAR has been, however, many times lower than that of the synaptic ones, based on immunogold labeling assessments [64] or studies of functional AMPAR using the two-photon uncaging of glutamate combined with non-stationary fluctuation analysis [65,67]. Estimates have shown an AMPAR density in immature Purkinje cells of approximately 910/μm^2^ at the synapses compared with 19/μm^2^ in the extrasynaptic membranes [65]. Similarly, the AMPAR count was several times less in the extrasynaptic membranes than at the synapses in the superficial DH [57,63]. Studies have also identified presynaptic AMPAR, both in the form of functional receptors [68] and as GluA1-4 immunoreactivity in primary afferent terminals [69]. The AMPAR subunit distribution revealed in the DH terminals closely resemble the distribution patterns of spinal GluA1-4, showing the predominant expression of presynaptic GluA4-containing CP-AMPAR in the laminae I–III (~70%), preferentially localized in terminals of unmyelinated (nociceptive) fibers, and the GluA2/A3 expression in the laminae III–IV, on myelinated fibers.

All four AMPAR subunits are highly homologous (~70% amino acid identity), having conserved transmembrane and extracellular domains, with only diverse C-terminal intracellular tails [70,71]. Most GluA2 subunits undergo RNA editing that replaces glutamine with arginine (Q/R editing) in the pore-forming region of the channel [72]; this ultimately prevents Ca^2+^-influx through the assembled channel [73]. Thus, almost all AMPAR in the adult brain (around 99%) are GluA2-containing, hence forming Ca^2+^-impermeable channels [74,75], whereas small amounts of GluA2-lacking AMPAR are Ca^2+^-permeable (CP). Consistently, AMPAR in the superficial DH are predominantly GluA2-containing (Ca^2+^-impermeable) channels. Electrophysiological studies have confirmed this by recording a weak blocking effect of intracellular polyamines on the AMPAR-mediated currents (i.e., an almost linear *I–V* curve) induced by pharmacological activation (total AMPAR pool) in the lamina II DH neurons [63,76]. Meanwhile, the postsynaptic AMPAR at nociceptive synapses – between primary nociceptive afferents and the DH neurons – consist of a mixed population of GluA2-containing (Ca^2+^-impermeable) receptors, with a relatively large proportion of GluA2-lacking, CP-AMPAR. This appears due to the compelling blocking effects of i) intracellular polyamines (i.e., the inwardly rectified *I–V* curve) and ii) selective antagonists of CP-AMPAR on the excitatory postsynaptic AMPAR-mediated currents induced by primary nociceptive afferent stimulation (EPSC) [22,34,77]. Aside from functional studies, quantitative estimates of the GluA1-labeled particles at the synapses of the superficial DH were found to exceed those of GluA2 [63].

## Nociceptive-induced AMPAR trafficking: Broken recycling of GluA1/A2 and chronic pain

**Figure 1.**
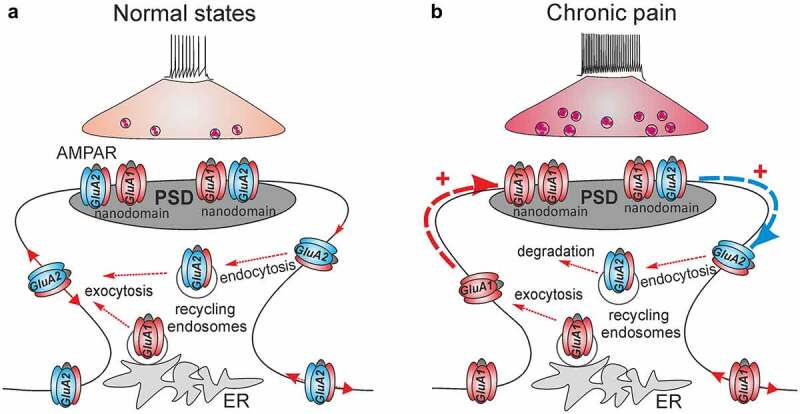
**Spinal AMPAR trafficking for nociceptive signaling in normal (healthy) and chronic pain states**. (a) After exocytosis from the intracellular stores such as the endoplasmic reticulum (ER), AMPAR laterally diffuse across the extrasynaptic plasma membrane to and from the postsynaptic density (PSD), undergoing constituent endocytosis/exocytosis cycles via recycling endosomes until are immobilized within the nanodomains of the receptors at PSD. (b) In chronic pain, the long-lasting activation of nociceptive afferents triggers the mobilization of the GluA2-containing AMPAR at PSD and the receptors undergo internalization from the synapses (blue arrow) followed by degradation (endocytosis via recycling endosomes). The GluA1-containing AMPAR replenish a pool of surface receptors and are mobilized to synapses (red arrow) to replace the GluA2-containing AMPAR within the nanodomains at PSD. Such a re-arrangement of the spinal AMPAR of different subunit compositions represents nociceptive plasticity in central pain pathways for the chronic pain maintenance

AMPAR are highly dynamic and mobile in plasma membrane: the receptors shuttle into and out of synapses and diffuse laterally (Figure 1a). AMPAR diffuse almost freely due to Brownian forces, either individually or within a cluster, before being trapped at the synapses. This is how a large fraction of AMPAR is recruited from their extrasynaptic pool to synapses for canonical LTP and hippocampal learning [78,79]. Another route for recruiting AMPAR is exocytosis of the receptors from the intracellular stores via recycling endosomes transporting AMPAR to the plasma membrane [80–82].

Nociceptive stimuli driven by long-lasting afferent activation break down the constituent recycling of spinal AMPAR. Experimental findings have made clear that the nociceptive-induced changes in dynamic AMPAR trafficking in the superficial DH neurons relate to the maintenance of persistent pain of different origins. Molecular biology studies have documented a rapid increase in the amount of GluA1, and a decrease in GluR2 in the plasma membrane (surface fraction) induced by peripheral injection of an inflammatory agent (complete Freund’s adjuvant, CFA); both changes were persistent over the time course of long-lasting pain in the CFA-induced model of persistent peripheral inflammation [23,34,63]. A similar re-distribution of GluA1-containing CP-AMPAR, namely, an increased number of GluA1 in the plasma membrane while a decrease in the cytosol, has been observed in different animal models of pain, such as neurogenic peripheral pain induced by capsaicin [83,84], and inflammatory pain induced by carrageenan [85] or formalin [86]. The nociceptive-induced upregulation of CP-AMPAR (GluA2-lacking receptors) in the superficial DH neurons was further confirmed through recordings of the augmented AMPAR-mediated currents, either alone or in combination with Ca^2+^ imaging technique, in persistent inflammatory pain [30,63,76], or following acute noxious stimulation [84] or after being induced by proinflammatory mediators [87,88].

Nociceptive stimuli facilitate the membrane insertion of GluA1-containing AMPAR at the extrasynaptic sites (Figure 1b). In the case of persistent inflammatory pain, the relative ratio of GluA1 at the postsynaptic and extrasynaptic membranes was estimated as 0.62 and 2.54, respectively, and 0.79 in the cytoplasm [63]. Neuroinflammatory mediators also enhanced the recycling of GluA1 homomers from the cytosol back to the extrasynaptic membrane, thereby promoting lateral diffusion of GluA1/GluA2 heteromers to the synapses [89,90].

However, increasing the number of GluA1-containing CP-AMPAR in the plasma membrane is insufficient to potentiate synapses [91]. The newly added receptors shuttling into the synapses have to be trapped and stabilized at postsynaptic density (PSD); this occurs via the interaction of an individual AMPAR with the scaffold proteins. Elegant approaches combined with super-resolution imaging for single-molecule localization and tracking have enabled the visualization of AMPAR in hippocampal neurons. Their stabilization inside synapses, along with the receptors’ compactness within nanodomains, was reversibly regulated by the expression level of the scaffold protein PSD-95 [92], or the auxiliary subunit stargazin which binds to PDZ domain scaffolds [91,93]. Electrophysiological recordings of the postsynaptic AMPAR-mediated EPSC in the lamina II DH neurons have documented the nociceptive-induced upregulation of CP-AMPAR at nociceptive synapses in chronic inflammatory pain conditions [19,34,77]. Such upregulation can last up to 21 days after the induction of inflammatory pain [94]. A rapid increase in GluA1, but not GluA2/A3, in the synaptosomal fraction [83] and at the nociceptive synapses established by C-fibers [84], was also confirmed in a model of capsaicin pain. Changes in the trafficking of spinal AMPAR in persistent pain conditions include the complementary boosted internalization of GluA2-containing, Ca^2+^-impermeable AMPAR from the synapses between the nociceptive afferents and the DH neurons (Figure 1b). Experimental evidence consists of electrophysiological recordings showing the inwardly rectified (GluA2-lacking) AMPAR-mediated EPSC in the lamina II DH neurons [19,34,77] and the quantification of the relative ratio of GluA2 across cellular compartments, which dropped to 0.61 at synapses, while increased to 1.2 in the cytoplasm, as rapidly as within 24 h of the onset of inflammatory pain [63]. Studies utilizing the latest advances involving super-resolution imaging in the superficial DH are in high demand to provide new insights into the dynamic re-distribution of AMPAR following nociceptive stimuli for single-particle tracking with nanoscale resolution. Using such an approach has allowed, to date, the identification of the GluA1 nanodomains at nociceptive terminals on peptidergic afferent fibers in the conditions of inflammatory pain [95]. However, whether it mirrors the organization and the distribution of the nanodomains of the receptors at the postsynaptic membrane at nociceptive synapses remains enigmatic. Likewise, the trafficking of spinal AMPAR of various subunit composition regarding different neuronal subtypes is still one of the major outstanding questions in normal states, let alone in cases of chronic pain.

## AMPAR phosphorylation as a molecular mechanism for broken trafficking

The phosphorylation of AMPAR subunit(s) is a key regulatory factor that determines not only biophysical properties but also the trafficking of the receptors, leading to the modulation of synaptic plasticity. GluA1 and/or GluA2 can undergo phosphorylation at different substrates (amino acid residues) at the C-tail, which contains multiple phosphorylation sites and protein-binding motifs. Phosphorylated receptors thus exhibit changes in single-channel properties [96] and interaction with scaffold proteins [96,97].

The mechanistic basis of the internalization of GluA2-containing AMPAR from nociceptive synapses, as deciphered by Park et al. [34], includes the phosphorylation of GluA2 at Serine (S) 880 by the protein kinase C (PKC) – the most heavily studied protein kinase – in the postsynaptic compartments of DH neurons; it disrupts the binding of the receptor to synaptic proteins, such as the AMPAR-binding protein (ABP)/glutamate receptor-interacting protein (GRIP), stargazing, and protein interacting with C kinase 1 (PICK1) [98]. The phosphorylated receptor consequently becomes capable of diffusing and shuttling out the synapse. The upstream trigger in this mechanism has been the activation of PKC subtype α, which is Ca^2+^-dependent process, induced by the activation of postsynaptic NMDA receptors (NMDAR), thereby  NMDAR-mediated Ca^2+^ influx, following the neuronal depolarization by nociceptive afferent stimuli [34]. In summary, the PKCα-dependent phosphorylation of GluA2 sets off a cascade of intracellular events, resulting in GluA2 internalization from nociceptive synapses, and eventually pain hypersensitivity [14]. Using the targeted mutation of the GluA2 phosphorylation site (by editing S882 to alanine, K882A) prevents the phosphorylation of GluA2 at S880 by PKC, and alleviates pain hypersensitivity in animals with persistent peripheral inflammation [34].

The phosphorylation of GluA1, at S831 and S845, has been found in neurogenic pain induced by capsaicin [59,99]. This was effectively blocked by the pharmacological inhibition of PKC [100]. Using genetic approaches (gene-silencing) combined with the spinal delivery of genetic materials, the PKCα-dependent upregulation of GluA1-containing CP-AMPAR has been validated in persistent inflammatory pain; moreover, the transient knockdown of spinal PKCα recovered both upregulated AMPAR-mediated currents and Ca^2+^ influx in the lamina II DH neurons, returning them back to normal levels [76] and alleviating persistent inflammatory pain [22].

Among other PKC isoforms, PKC gamma was found to be capable of phosphorylating GluA1 at S831, but not at S845, in cases of neuropathic pain [101], and to enhance the membrane insertion of GluA1 in post-surgical pain [102]. In addition to S831 and S845, the other substrate on the GluA1 subunit – the S818 phosphorylation site (the PKC substrate only) – has been found to be critical for the synaptic incorporation of GluA1-containing CP-AMPAR, and the hippocampal LTP [103]. However, the implications of this highly conserved GluA1 residue for nociceptive-induced trafficking of the receptors remains unknown.

Apart from PKC, other protein kinases also phosphorylate the GluA1 subunit. The role of protein kinase A (PKA) in promoting the membrane insertion of GluA1 is fairly notable. PKA targeted the S831 and S845 sites on the GluA1 in inflammatory pain [85] and capsaicin-induced pain models [104]. Meanwhile, the engagement of Ca^2+^-calmodulin-dependent kinase II (CaMKII) in the phosphorylation of spinal GluA1 remains, to some degree, debatable, with conflicting evidence across the literature. Some of the studies have reported no link between the phosphorylation of spinal GluA1, at S831 and/nor S845 sites, and the activation of CaMKII in chronic injury [101] or thermal pain models [105], while others have shown an increased expression of CaMKII that triggered the membrane insertion of GluA1 in a capsaicin pain model [83,106], as well as the CaMKII alpha-dependent GluA1 phosphorylation at S831 in postoperative pain [107]. In comparison, the insertion of CP-AMPAR into the synapses in the brain was found to depend upon CaMK activation [108], while the CaMKII-dependent phosphorylation of GluA1 at S831 clearly potentiated synaptic transmission [109]. Consequently, knock-in mutations in GluA1 phosphorylation sites targeted by CaMKII and PKA produced deficits in hippocampal LTP and unambiguous memory defects [110], indicating that the PKA-driven phosphorylation of GluA1 is not sufficient for the receptors’ incorporation into synapses, but requires also CaMKII activation [111].

## Perturbed trafficking of spinal AMPAR in chronic pain: To what degree does it underlie nociceptive plasticity in the DH?

**Figure 2.**
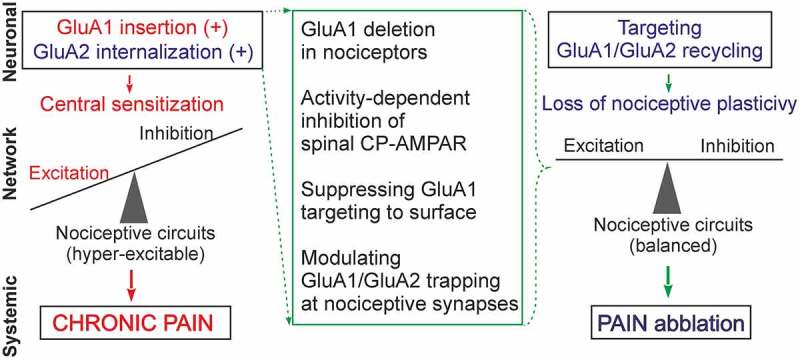
Nociceptive-induced changes in the trafficking of spinal AMPAR (neuronal level) cause a broken balance between synaptic excitation and inhibition that leads to overall hyper-excitability and central sensitization of the DH (network level); aberrant signaling in central pain pathways turns pain chronic (systemic level). Various scenarios for targeting the broken GluA1 and/or GluA2 recycling can be implemented to suppress the AMPAR-mediated nociceptive plasticity, and recover the relative balance between neuronal excitation and inhibition in the DH circuits, thereby alleviating chronic pain

Nociceptive-induced changes in functional AMPAR lead to the altered excitability of the DH neurons; they become over-excited, causing overall hyperexcitability of the DH, a state that is associated with chronic pain in persistent peripheral inflammation [112], or after spinal cord injury [89,113]. The re-distribution of spinal AMPAR across neuronal compartments implies nociceptive neural plasticity evoked by a long-lasting presynaptic drive from nociceptive afferents. This AMPAR-mediated plasticity largely contributes to central sensitization and pain hypersensitivity (Figure 2).

Nociceptive-induced changes in spinal AMPAR appear to be cell-type specific. A prominent upregulation of CP-AMPAR has been documented in lamina II DH neurons characterized by intrinsic tonic firing properties in persistent inflammatory pain [30,63,76]. Neurons exhibiting tonic firing properties are a mixed population of heterogeneous cell types, overlapping inhibitory and, to a lesser extent, excitatory interneurons [114,115]. Different patterns of GluA1-4 expression were detected in the superficial DH, even in normal conditions: a majority of GABA and NK1 receptor‐positive neurons expressed CP-AMPAR [116], with a major GluA1-immunoreactivity (78%) identified for inhibitory lamina I–II neurons, while almost all GluR2/3-immunoreactivity (97%) was identified for excitatory neurons [117].

Electrophysiological studies have revealed a shift in the relative balance between synaptic excitation and inhibition within the superficial DH, by documenting an increased synaptic excitation accompanied by a decreased synaptic inhibition that same neurons receive in persistent inflammatory pain [112] and after spinal cord injury [113]. The disrupted balance in terms of boosted neuronal excitation leads to nociceptive circuits becoming hyper-excitable and involved in aberrant sensory processing (Figure 2). Although the identity of neurons with perturbed AMPAR trafficking remains largely unclear, the excitatory drive was found to be impaired in GABAergic neurons in case of neuropathic pain [118]. The loss of GABAergic inhibition in the superficial DH has been established in pain states of a diverse nature: neuropathic [119], injury-induced [120,121] or inflammatory pain [122]. A recent study has revealed that such a loss may be due to a loss of GABAergic interneurons [123], suggesting a morphological basis for the disrupted balance between synaptic excitation and inhibition in chronic pain.

The mechanisms of nociceptive plasticity in the DH circuits also include presynaptic changes. It has been shown that ectopic firing by primary nociceptive afferents is associated with the enhanced release of glutamate [124,125]. Thus, boosted release, often pertaining to “spillover”, prompts the activation of perisynaptic/extrasynaptic receptors. In such a scenario, extrasynaptic glutamate escapes from the nociceptive terminals lead to the activation of an amount of extrasynaptic AMPAR, which are highly expressed in the lamina I–II DH neurons. The activation of extrasynaptic AMPAR consolidates changes in the postsynaptic AMPAR-mediated transmission, and amplifies the hyper-activation of the DH neurons, causing over-excitation within nociceptive circuits. Studies of the brain circuits over the last few decades have postulated an increased release probability in LTP [47,126,127]. The implementation of recent advances in genetically engineered optical sensors for the detection of glutamate release at the single synapse level would enable the detection of changes in the quantal content of glutamate released by nociceptive afferents in cases of chronic pain.

The extrasynaptic escape of glutamate plays an important role in inter-synaptic cross-talk [128], potentially boosting robust cross-talk between nociceptive synapses and their neighbors within highly interconnected DH circuits. Furthermore, an elegant hypothesis of “silent synapses” depicts the AMPAR role in synaptic plasticity in the brain [129,130], serving as a form of morphological plasticity, in which a proportion of synapses with absent or nonfunctional AMPAR in the superficial DH neurons can be switched on upon a long-lasting nociceptive afferent drive. In addition to the nociceptive-driven subcellular re-arrangement of AMPAR, such a morphological re-arrangement between non-active and active synapses would extend nociceptive signaling, and lead to an increase in pain hypersensitivity due to a complex of changes in the central pain pathways.

## Would targeting spinal AMPAR provide relief in chronic pain without side effects? Challenges and perspectives

Up-to-date knowledge of broken AMPAR trafficking as a cellular mechanism that turns pain chronic in central sensory pathways has identified new therapeutic targets and favors developing target-based strategies, which can be predicted as an effective treatment against chronic pain. The current understanding of deciphered signaling cascades highlights a critical step – potentially best suited for reliable alleviation of chronic pain – that focuses on restoring the broken GluA1/GluA2 recycling in the superficial DH neurons to a normal state. A crucial role that GluA1-containing CP-AMPAR plays in LTP and synaptic plasticity precludes the deletion of GluA1 (in order to prevent systemic side effects), but it can be applicable when utilized exclusively in nociceptive pathways (Figure 2). Such an approach – the deletion of GluA1 in primary nociceptors – results in the ablation of GluA1-containing AMPAR in the DH nociceptive terminals, and reduces mechanical hypersensitivity in mice with chronic pain of an inflammatory or arthritic origin [17]; no changes in pain sensation were found after a similar deletion of GluA2. A major challenge, however, remains with regard to suppressing the activity-induced upregulation of GluA1-containing CP-AMPAR in the superficial DH neurons.

Several strategies focusing on spinal AMPAR have been probed in parallel. Experimental attempts comprise, as of now, a number of different strategies. These can be subdivided into two main directions: pharmacological or genetic modulation of the GluA1, and/or GluA2 recycling. Amongst the promising pharmacological approaches is the combination of the advantages of using activity-dependent antagonists of CP-AMPAR, such as dicationic compounds, with localized spinal administration (e.g., intrathecal delivery to a targeted spinal cord region) [31]. The exact mechanism of activity-dependent inhibition has been described in terms of the exceptional ability of dicationic compounds to modulate glutamatergic transmission in the brain [131,132] and to restore the nociceptive-induced upregulation of AMPAR-mediated currents in lamina II DH neurons in persistent inflammatory pain [63,76]. In rodents, dicationic compounds reduced hyperalgesia and shortened the period of inflammatory pain maintenance [31]. Using various tests by addressing potential changes in the animals’ sensitivity to thermal or mechanical stimuli, locomotion activity, or exploratory behavior (anxiety), no adverse effects were observed following treatment.

Novel therapeutic strategies have focused on targeting the broken GluA1/GluA2 recycling via interfering with intracellular signaling cascades. One of those can act to suppress the upstream trigger, which is the activation of PKCα. The pharmacological inhibition of spinal PKCα (using a selective PKCα inhibitor peptide, C2-4) or genetic inhibition of PKCα (with antisense oligonucleotides) has both effectively reduced nociceptive hypersensitivity in animals, and also provided relief in pain-induced locomotive deficit and anxiety in persistent peripheral inflammation [22,76]. At the cellular level, therapeutic effects were manifested in the form of restored AMPAR-mediated currents in the lamina II DH neurons and at nociceptive synapses [22,76]. Targeting scaffold proteins regulating the postsynaptic trapping of AMPAR at PSD can be another alternative (Figure 2). This has been recently validated using an engineered peptide inhibitor of PICK1 to alleviate mechanical hyperalgesia in injury-induced neuropathic pain [133]. Likewise, the peptide GluA2-3y, which inhibits endocytosis of GluA2-containing AMPAR, revealed an antinociceptive effect on neuropathic pain [134]. Targeting transmembrane AMPAR regulatory proteins (TARP), whose specific expression patterns were identified in the superficial DH [135], seemed a promising direction for modulating CP-AMPAR-mediated plasticity at nociceptive synapses, prone to fewer side effects. A recent study has suggested selective TARP *γ-*8 blockage for the treatment of chronic pain [136].

In conclusion, the ongoing development of reliable and well-predictable strategies against chronic pain, further to the current approaches and directions noted here, remains active. Emerging advances have been made for targeted treatment options based on the recently discovered signaling cascades and newly identified proteins regulating AMPAR trafficking; this will help focus on refining approaches for fine-tuning the AMPAR-mediated nociceptive plasticity in central pain pathways in such a way to identify the most powerful therapies against chronic pain, with great care being taken to reduce (or prevent) any side effects.

## References

[1] Collaborators G. Global, regional, and national incidence, prevalence, and years lived with disability for 301 acute and chronic diseases and injuries in 188 countries, 1990-2013: a systematic analysis for the global burden of disease study 2013. Lancet. 2015 8 22;386(9995):743–800. 26063472 10.1016/S0140-6736(15)60692-4 PMC4561509

[2] Breivik H, Collett B, Ventafridda V, et al. Survey of chronic pain in Europe: prevalence, impact on daily life, and treatment. Eur J Pain. 2006 5;10(4):287–333. 16095934 10.1016/j.ejpain.2005.06.009

[3] Rice AS, Smith BH, Blyth FM. Pain and the global burden of disease. Pain. 2016 4;157(4):791–796. 26670465 10.1097/j.pain.0000000000000454

[4] Dahlhamer J, Lucas J, Zelaya C, et al. Prevalence of chronic pain and high-impact chronic pain among adults – United States, 2016. Morbidity Mortality Weekly Rep. 2018;67(36):1001–1006. 10.15585/mmwr.mm6736a2 PMC6146950 30212442

[5] Goldberg DS, McGee SJ. Pain as a global public health priority. BMC Public Health. 2011 10;6(11):770. 10.1186/1471-2458-11-770 PMC3201926 21978149

[6] Treede RD, Rief W, Barke A, et al. Chronic pain as a symptom or a disease: the IASP classification of chronic pain for the international classification of diseases (ICD-11). Pain. 2019 1;160(1):19–27. 30586067 10.1097/j.pain.0000000000001384

[7] Scholz J, Finnerup NB, Attal N, et al. The IASP classification of chronic pain for ICD-11: chronic neuropathic pain. Pain. 2019 1;160(1):53–59. 30586071 10.1097/j.pain.0000000000001365 PMC6310153

[8] Peirs C, Seal RP. Neural circuits for pain: recent advances and current views. Science. 2016 11 4;354(6312):578–584. 27811268 10.1126/science.aaf8933 PMC11327866

[9] Kuner R. Central mechanisms of pathological pain. Nat Med. 2010 11;16(11):1258–1266. 20948531 10.1038/nm.2231

[10] Bushnell MC, Ceko M, Low LA. Cognitive and emotional control of pain and its disruption in chronic pain. Nat Rev Neurosci. 2013 7;14(7):502–511. 23719569 10.1038/nrn3516 PMC4465351

[11] Chater TE, Goda Y. The role of AMPA receptors in postsynaptic mechanisms of synaptic plasticity. Front Cell Neurosci. 2014;8:401. 25505875 10.3389/fncel.2014.00401 PMC4245900

[12] Henley JM, Wilkinson KA. Synaptic AMPA receptor composition in development, plasticity and disease. Nat Rev Neurosci. 2016 6;17(6):337–350. 27080385 10.1038/nrn.2016.37

[13] Beneyto M, Meador-Woodruff JH. Expression of transcripts encoding AMPA receptor subunits and associated postsynaptic proteins in the macaque brain. J Comp Neurol. 2004 1 19;468(4):530–554. 14689485 10.1002/cne.10981

[14] Kopach O, Extrasynaptic VN. AMPA receptors in the dorsal horn: evidence and functional significance. Brain Res Bull. 2013 4;93:47–56. 23194665 10.1016/j.brainresbull.2012.11.004

[15] Tao YX. AMPA receptor trafficking in inflammation-induced dorsal horn central sensitization. Neurosci Bull. 2012 4;28(2):111–120. 22466122 10.1007/s12264-012-1204-z PMC3324122

[16] Qiu S, Zhang M, Liu Y, et al. GluA1 phosphorylation contributes to postsynaptic amplification of neuropathic pain in the insular cortex. J Neurosci. 2014 10 1;34(40):13505–13515. 25274827 10.1523/JNEUROSCI.1431-14.2014 PMC4340970

[17] Gangadharan V, Wang R, Ulzhöfer B, et al. Peripheral calcium-permeable AMPA receptors regulate chronic inflammatory pain in mice. J Clin Invest. 2011 4;121(4):1608–1623. 21383497 10.1172/JCI44911 PMC3069784

[18] Garry EM, Moss A, Rosie R, et al. Specific involvement in neuropathic pain of AMPA receptors and adapter proteins for the GluR2 subunit. Mol Cell Neurosci. 2003 9;24(1):10–22. 14550765 10.1016/s1044-7431(03)00134-9

[19] Katano T, Furue H, Okuda-Ashitaka E, et al. N-ethylmaleimide-sensitive fusion protein (NSF) is involved in central sensitization in the spinal cord through GluR2 subunit composition switch after inflammation. Eur J Neurosci. 2008 6;27(12):3161–3170. 18598260 10.1111/j.1460-9568.2008.06293.x

[20] Latremoliere A, Woolf CJ. Central sensitization: a generator of pain hypersensitivity by central neural plasticity. J Pain. 2009 9;10(9):895–926. 19712899 10.1016/j.jpain.2009.06.012 PMC2750819

[21] Woolf CJ, Salter MW. Neuronal plasticity: increasing the gain in pain. Science. 2000 6 9;288(5472):1765–1769. 10846153 10.1126/science.288.5472.1765

[22] Kopach O, Krotov V, Shysh A, et al. Spinal PKCα inhibition and gene-silencing for pain relief: AMPAR trafficking at the synapses between primary afferents and sensory interneurons. Sci Rep. 2018 7 6;8(1):10285. 29980697 10.1038/s41598-018-28512-9 PMC6035211

[23] Park JS, Yaster M, Guan X, et al. Role of spinal cord alpha-amino-3-hydroxy-5-methyl-4-isoxazolepropionic acid receptors in complete Freund’s adjuvant-induced inflammatory pain. Mol Pain. 2008 12 30;4:67. 19116032 10.1186/1744-8069-4-67 PMC2628655

[24] Sorkin LS, Yaksh TL, Doom CM. Mechanical allodynia in rats is blocked by a Ca2+ permeable AMPA receptor antagonist. Neuroreport. 1999 11 26;10(17):3523–3526. 10619637 10.1097/00001756-199911260-00011

[25] Gwak YS, Kang J, Leem JW, et al. Spinal AMPA receptor inhibition attenuates mechanical allodynia and neuronal hyperexcitability following spinal cord injury in rats. J Neurosci Res. 2007 8 15;85(11):2352–2359. 17549753 10.1002/jnr.21379

[26] Chen SR, Zhou HY, Byun HS, et al. Nerve injury increases GluA2-lacking AMPA receptor prevalence in spinal cords: functional significance and signaling mechanisms. J Pharmacol Exp Ther. 2013 12;347(3):765–772. 24030012 10.1124/jpet.113.208363 PMC3836313

[27] Jin HC, Keller AJ, Jung JK, et al. Epidural tezampanel, an AMPA/kainate receptor antagonist, produces postoperative analgesia in rats. Anesth Analg. 2007 10;105(4):1152–1159. table of contents. 17898404 10.1213/01.ane.0000281435.58012.e3

[28] Lee HJ, Pogatzki-Zahn EM, Brennan TJ. The effect of the AMPA/kainate receptor antagonist LY293558 in a rat model of postoperative pain. J Pain. 2006 10;7(10):768–777. 17018337 10.1016/j.jpain.2006.03.010

[29] Zahn PK, Pogatzki-Zahn EM, Brennan TJ. Spinal administration of MK-801 and NBQX demonstrates NMDA-independent dorsal horn sensitization in incisional pain. Pain. 2005 4;114(3):499–510. 15777875 10.1016/j.pain.2005.01.018

[30] Kopach O, Viatchenko-Karpinski V, Belan P, et al. Development of inflammation-induced hyperalgesia and allodynia is associated with the upregulation of extrasynaptic AMPA receptors in tonically firing lamina II dorsal horn neurons. Front Physiol. 2012;3:391. 23060815 10.3389/fphys.2012.00391 PMC3462428

[31] Kopach O, Krotov V, Goncharenko J, et al. Inhibition of spinal Ca(2+)-permeable AMPA receptors with dicationic compounds alleviates persistent inflammatory pain without adverse effects. Front Cell Neurosci. 2016;10:50. 26973464 10.3389/fncel.2016.00050 PMC4770326

[32] Hartmann B, Ahmadi S, Heppenstall PA, et al. The AMPA receptor subunits GluR-A and GluR-B reciprocally modulate spinal synaptic plasticity and inflammatory pain. Neuron. 2004 11 18;44(4):637–650. 15541312 10.1016/j.neuron.2004.10.029

[33] Zhang B, Tao F, Liaw WJ, et al. Effect of knock down of spinal cord PSD-93/chapsin-110 on persistent pain induced by complete Freund’s adjuvant and peripheral nerve injury. Pain. 2003 11;106(1–2):187–196. 14581127 10.1016/j.pain.2003.08.003

[34] Park JS, Voitenko N, Petralia RS, et al. Persistent inflammation induces GluR2 internalization via NMDA receptor-triggered PKC activation in dorsal horn neurons. J Neurosci. 2009 3 11;29(10):3206–3219. 19279258 10.1523/JNEUROSCI.4514-08.2009 PMC2664544

[35] Youn DH, Royle G, Kolaj M, et al. Enhanced LTP of primary afferent neurotransmission in AMPA receptor GluR2-deficient mice. Pain. 2008 5;136(1–2):158–167. 17826911 10.1016/j.pain.2007.07.001

[36] Jia Z, Agopyan N, Miu P, et al. Enhanced LTP in mice deficient in the AMPA receptor GluR2. Neuron. 1996 11;17(5):945–956. 8938126 10.1016/s0896-6273(00)80225-1

[37] Brusa R, Zimmermann F, Koh DS, et al. Early-onset epilepsy and postnatal lethality associated with an editing-deficient GluR-B allele in mice. Science. 1995 12 8;270(5242):1677–1680. 7502080 10.1126/science.270.5242.1677

[38] Lamsa KP, Heeroma JH, Somogyi P, et al. Anti-Hebbian long-term potentiation in the hippocampal feedback inhibitory circuit. Science. 2007 3 2;315(5816):1262–1266. 17332410 10.1126/science.1137450 PMC3369266

[39] Meng Y, Zhang Y, Jia Z. Synaptic transmission and plasticity in the absence of AMPA glutamate receptor GluR2 and GluR3. Neuron. 2003 7 3;39(1):163–176. 12848940 10.1016/s0896-6273(03)00368-4

[40] Zamanillo D, Sprengel R, Hvalby O, et al. Importance of AMPA receptors for hippocampal synaptic plasticity but not for spatial learning. Science. 1999 6 11;284(5421):1805–1811. 10364547 10.1126/science.284.5421.1805

[41] Bannerman DM, Borchardt T, Jensen V, et al. Somatic accumulation of GluA1-AMPA receptors leads to selective cognitive impairments in mice. Front Mol Neurosci. 2018;11:199. 29988555 10.3389/fnmol.2018.00199 PMC6026654

[42] Reisel D, Bannerman DM, Schmitt WB, et al. Spatial memory dissociations in mice lacking GluR1. Nat Neurosci. 2002 9;5(9):868–873. 12195431 10.1038/nn910

[43] Schmitt WB, Deacon RM, Seeburg PH, et al. A within-subjects, within-task demonstration of intact spatial reference memory and impaired spatial working memory in glutamate receptor-A-deficient mice. J Neurosci. 2003 5 1;23(9):3953–3959. 12736365 10.1523/JNEUROSCI.23-09-03953.2003 PMC6742186

[44] Ikeda H, Heinke B, Ruscheweyh R, et al. Synaptic plasticity in spinal lamina I projection neurons that mediate hyperalgesia. Science. 2003 2 21;299(5610):1237–1240. 12595694 10.1126/science.1080659

[45] Randić M, Jiang MC, Cerne R. Long-term potentiation and long-term depression of primary afferent neurotransmission in the rat spinal cord. J Neurosci. 1993 12;13(12):5228–5241. 8254370 10.1523/JNEUROSCI.13-12-05228.1993 PMC6576415

[46] Henneberger C, Bard L, Panatier A, et al. LTP induction boosts glutamate spillover by driving withdrawal of perisynaptic astroglia. Neuron. 2020 Dec;108(5):919-936. 32976770 10.1016/j.neuron.2020.08.030 PMC7736499

[47] Kopach O, Zheng K, Rusakov DA. Optical monitoring of glutamate release at multiple synapses in situ detects changes following LTP induction. Mol Brain. 2020 3 13;13(1):39. 32169106 10.1186/s13041-020-00572-x PMC7071671

[48] Ikeda H, Stark J, Fischer H, et al. Synaptic amplifier of inflammatory pain in the spinal dorsal horn. Science. 2006 6 16;312(5780):1659–1662. 16778058 10.1126/science.1127233

[49] Understanding SJ. LTP in pain pathways. Mol Pain. 2007 4;3(3):9. 17407590 10.1186/1744-8069-3-9 PMC1852298

[50] Zhang XC, Zhang YQ, Zhao ZQ. Involvement of nitric oxide in long-term potentiation of spinal nociceptive responses in rats. Neuroreport. 2005 8 1;16(11):1197–1201. 16012348 10.1097/00001756-200508010-00013

[51] Isaac JT, Ashby MC, McBain CJ. The role of the GluR2 subunit in AMPA receptor function and synaptic plasticity. Neuron. 2007 6 21;54(6):859–871. 17582328 10.1016/j.neuron.2007.06.001

[52] Lu W, Shi Y, Jackson AC, et al. Subunit composition of synaptic AMPA receptors revealed by a single-cell genetic approach. Neuron. 2009 4 30;62(2):254–268. 19409270 10.1016/j.neuron.2009.02.027 PMC3632349

[53] Wenthold RJ, Petralia RS, Blahos J II, et al. Evidence for multiple AMPA receptor complexes in hippocampal CA1/CA2 neurons. J Neurosci. 1996 3 15;16(6):1982–1989. 8604042 10.1523/JNEUROSCI.16-06-01982.1996 PMC6578515

[54] Pelkey KA, Barksdale E, Craig MT, et al. Pentraxins coordinate excitatory synapse maturation and circuit integration of parvalbumin interneurons. Neuron. 2015 3 18;85(6):1257–1272. 25754824 10.1016/j.neuron.2015.02.020 PMC4368480

[55] Zhu JJ, Esteban JA, Hayashi Y, et al. Postnatal synaptic potentiation: delivery of GluR4-containing AMPA receptors by spontaneous activity. Nat Neurosci. 2000 11;3(11):1098–1106. 11036266 10.1038/80614

[56] Engelman HS, Allen TB, MacDermott AB. The distribution of neurons expressing calcium-permeable AMPA receptors in the superficial laminae of the spinal cord dorsal horn. J Neurosci. 1999 3 15;19(6):2081–2089. 10066261 10.1523/JNEUROSCI.19-06-02081.1999 PMC6782571

[57] Petralia RS, Wang YX, Mayat E, et al. Glutamate receptor subunit 2-selective antibody shows a differential distribution of calcium-impermeable AMPA receptors among populations of neurons. J Comp Neurol. 1997 9 1;385(3):456–476. 9300771 10.1002/(sici)1096-9861(19970901)385:3<456::aid-cne9>3.0.co;2-2

[58] Todd AJ. Neuronal circuitry for pain processing in the dorsal horn. Nat Rev Neurosci. 2010 12;11(12):823–836. 21068766 10.1038/nrn2947 PMC3277941

[59] Nagy GG, Al-Ayyan M, Andrew D, et al. Widespread expression of the AMPA receptor GluR2 subunit at glutamatergic synapses in the rat spinal cord and phosphorylation of GluR1 in response to noxious stimulation revealed with an antigen-unmasking method. J Neurosci. 2004 6 23;24(25):5766–5777. 15215299 10.1523/JNEUROSCI.1237-04.2004 PMC6729210

[60] Polgár E, Watanabe M, Hartmann B, et al. Expression of AMPA receptor subunits at synapses in laminae I-III of the rodent spinal dorsal horn. Mol Pain. 2008 1;23(4):5. 10.1186/1744-8069-4-5 PMC2248168 18215271

[61] Brown KM, Wrathall JR, Yasuda RP, et al. Quantitative measurement of glutamate receptor subunit protein expression in the postnatal rat spinal cord. Brain Res Dev Brain Res. 2002 8 30;137(2):127–133. 12220704 10.1016/s0165-3806(02)00435-2

[62] Nichols ML, Allen BJ, Rogers SD, et al. Transmission of chronic nociception by spinal neurons expressing the substance P receptor. Science. 1999 11 19;286(5444):1558–1561. 10567262 10.1126/science.286.5444.1558

[63] Kopach O, Kao SC, Petralia RS, et al. Inflammation alters trafficking of extrasynaptic AMPA receptors in tonically firing lamina II neurons of the rat spinal dorsal horn. Pain. 2011 4;152(4):912–923. 21282008 10.1016/j.pain.2011.01.016 PMC3079375

[64] Masugi-Tokita M, Tarusawa E, Watanabe M, et al. Number and density of AMPA receptors in individual synapses in the rat cerebellum as revealed by SDS-digested freeze-fracture replica labeling. J Neurosci. 2007 2 21;27(8):2135–2144. 17314308 10.1523/JNEUROSCI.2861-06.2007 PMC6673557

[65] Tanaka J, Matsuzaki M, Tarusawa E, et al. Number and density of AMPA receptors in single synapses in immature cerebellum. J Neurosci. 2005 1 26;25(4):799–807. 15673659 10.1523/JNEUROSCI.4256-04.2005 PMC6725634

[66] Matsuzaki M, Ellis-Davies GC, Nemoto T, et al. Dendritic spine geometry is critical for AMPA receptor expression in hippocampal CA1 pyramidal neurons. Nat Neurosci. 2001 11;4(11):1086–1092. 11687814 10.1038/nn736 PMC4229049

[67] Momiyama A, Silver RA, Hausser M, et al. The density of AMPA receptors activated by a transmitter quantum at the climbing fibre-Purkinje cell synapse in immature rats. J Physiol. 2003 5 15;549(Pt 1):75–92. 12665613 10.1113/jphysiol.2002.033472 PMC2342931

[68] Lu CR, Hwang SJ, Phend KD, et al. Primary afferent terminals in spinal cord express presynaptic AMPA receptors. J Neurosci. 2002 11 1;22(21):9522–9529. 12417676 10.1523/JNEUROSCI.22-21-09522.2002 PMC6758021

[69] Lee CJ, Bardoni R, Tong CK, et al. Functional expression of AMPA receptors on central terminals of rat dorsal root ganglion neurons and presynaptic inhibition of glutamate release. Neuron. 2002 7 3;35(1):135–146. 12123614 10.1016/s0896-6273(02)00729-8

[70] Keinänen K, Wisden W, Sommer B, et al. A family of AMPA-selective glutamate receptors. Science. 1990 8 3;249(4968):556–560. 2166337 10.1126/science.2166337

[71] Sommer B, Keinänen K, Verdoorn TA, et al. Flip and flop: a cell-specific functional switch in glutamate-operated channels of the CNS. Science. 1990 9 28;249(4976):1580–1585. 1699275 10.1126/science.1699275

[72] Greger IH, Khatri L, Kong X, et al. AMPA receptor tetramerization is mediated by Q/R editing. Neuron. 2003 11 13;40(4):763–774. 14622580 10.1016/s0896-6273(03)00668-8

[73] Burnashev N, Monyer H, Seeburg PH, et al. Divalent ion permeability of AMPA receptor channels is dominated by the edited form of a single subunit. Neuron. 1992 1;8(1):189–198. 1370372 10.1016/0896-6273(92)90120-3

[74] Traynelis SF, Wollmuth LP, McBain CJ, et al. Glutamate receptor ion channels: structure, regulation, and function. Pharmacol Rev. 2010 9;62(3):405–496. 20716669 10.1124/pr.109.002451 PMC2964903

[75] Cull-Candy S, Kelly L, Farrant M. Regulation of Ca2+-permeable AMPA receptors: synaptic plasticity and beyond. Curr Opin Neurobiol. 2006 6;16(3):288–297. 16713244 10.1016/j.conb.2006.05.012

[76] Kopach O, Viatchenko-Karpinski V, Atianjoh FE, et al. PKCα is required for inflammation-induced trafficking of extrasynaptic AMPA receptors in tonically firing lamina II dorsal horn neurons during the maintenance of persistent inflammatory pain. J Pain. 2013 2;14(2):182–192. 23374940 10.1016/j.jpain.2012.10.015 PMC3564511

[77] Vikman KS, Rycroft BK, Christie MJ. Switch to Ca2+-permeable AMPA and reduced NR2B NMDA receptor-mediated neurotransmission at dorsal horn nociceptive synapses during inflammatory pain in the rat. J Physiol. 2008 1 15;586(2):515–527. 18033811 10.1113/jphysiol.2007.145581 PMC2375596

[78] Choquet D, Triller A. The role of receptor diffusion in the organization of the postsynaptic membrane. Nat Rev Neurosci. 2003 4;Apr(4):251–265. 10.1038/nrn1077 12671642

[79] Penn AC, Zhang CL, Georges F, et al. Hippocampal LTP and contextual learning require surface diffusion of AMPA receptors. Nature. 2017 9 21;549(7672):384–388. 28902836 10.1038/nature23658 PMC5683353

[80] Makino H, Malinow R. AMPA receptor incorporation into synapses during LTP: the role of lateral movement and exocytosis. Neuron. 2009 11 12;64(3):381–390. 19914186 10.1016/j.neuron.2009.08.035 PMC2999463

[81] Park M, Penick EC, Edwards JG, et al. Recycling endosomes supply AMPA receptors for LTP. Science. 2004 9 24;305(5692):1972–1975. 15448273 10.1126/science.1102026

[82] Wu D, Bacaj T, Morishita W, et al. Postsynaptic synaptotagmins mediate AMPA receptor exocytosis during LTP. Nature. 2017 4 20;544(7650):316–321. 28355182 10.1038/nature21720 PMC5734942

[83] Galan A, Laird JM, Cervero F. In vivo recruitment by painful stimuli of AMPA receptor subunits to the plasma membrane of spinal cord neurons. Pain. 2004 12;112(3):315–323. 15561387 10.1016/j.pain.2004.09.011

[84] Larsson M, Broman J. Translocation of GluR1-containing AMPA receptors to a spinal nociceptive synapse during acute noxious stimulation. J Neurosci. 2008 7 9;28(28):7084–7090. 18614677 10.1523/JNEUROSCI.5749-07.2008 PMC6670484

[85] Choi JI, Svensson CI, Koehrn FJ, et al. Peripheral inflammation induces tumor necrosis factor dependent AMPA receptor trafficking and Akt phosphorylation in spinal cord in addition to pain behavior. Pain. 2010 5;149(2):243–253. 20202754 10.1016/j.pain.2010.02.008 PMC2860679

[86] Pezet S, Marchand F, D’Mello R, et al. Phosphatidylinositol 3-kinase is a key mediator of central sensitization in painful inflammatory conditions. J Neurosci. 2008 4 16;28(16):4261–4270. 18417706 10.1523/JNEUROSCI.5392-07.2008 PMC2935680

[87] Kohno T, Wang H, Amaya F, et al. Bradykinin enhances AMPA and NMDA receptor activity in spinal cord dorsal horn neurons by activating multiple kinases to produce pain hypersensitivity. J Neurosci. 2008 4 23;28(17):4533–4540. 18434532 10.1523/JNEUROSCI.5349-07.2008 PMC2653863

[88] Liu T, Jiang CY, Fujita T, et al. Enhancement by interleukin-1β of AMPA and NMDA receptor-mediated currents in adult rat spinal superficial dorsal horn neurons. Mol Pain. 2013 3 28;9:16. 23537341 10.1186/1744-8069-9-16 PMC3622562

[89] Ferguson AR, Christensen RN, Gensel JC, et al. Cell death after spinal cord injury is exacerbated by rapid TNF alpha-induced trafficking of GluR2-lacking AMPARs to the plasma membrane. J Neurosci. 2008 10 29;28(44):11391–11400. 18971481 10.1523/JNEUROSCI.3708-08.2008 PMC2598739

[90] Leonoudakis D, Zhao P, Beattie EC. Rapid tumor necrosis factor alpha-induced exocytosis of glutamate receptor 2-lacking AMPA receptors to extrasynaptic plasma membrane potentiates excitotoxicity. J Neurosci. 2008 2 27;28(9):2119–2130. 18305246 10.1523/JNEUROSCI.5159-07.2008 PMC6671833

[91] Schnell E, Sizemore M, Karimzadegan S, et al. Direct interactions between PSD-95 and stargazin control synaptic AMPA receptor number. Proc Natl Acad Sci U S A. 2002 10 15;99(21):13902–13907. 12359873 10.1073/pnas.172511199 PMC129795

[92] Nair D, Hosy E, Petersen JD, et al. Super-resolution imaging reveals that AMPA receptors inside synapses are dynamically organized in nanodomains regulated by PSD95. J Neurosci. 2013 8 7;33(32):13204–13224. 23926273 10.1523/JNEUROSCI.2381-12.2013 PMC6619720

[93] Opazo P, Labrecque S, Tigaret CM, et al. CaMKII triggers the diffusional trapping of surface AMPARs through phosphorylation of stargazin. Neuron. 2010 7 29;67(2):239–252. 20670832 10.1016/j.neuron.2010.06.007

[94] Taylor BK, Sinha GP, Donahue RR, et al. Opioid receptors inhibit the spinal AMPA receptor Ca(2+) permeability that mediates latent pain sensitization. Exp Neurol. 2019 4;314:58–66. 30660616 10.1016/j.expneurol.2019.01.003 PMC6559354

[95] Woodhams SG, Markus R, Gowler PRW, et al. Cell type-specific super-resolution imaging reveals an increase in calcium-permeable AMPA receptors at spinal peptidergic terminals as an anatomical correlate of inflammatory pain. Pain. 2019 11;160(11):2641–2650. 31425488 10.1097/j.pain.0000000000001672

[96] Banke TG, Greenwood JR, Christensen JK, et al. Identification of amino acid residues in GluR1 responsible for ligand binding and desensitization. J Neurosci. 2001 5 1;21(9):3052–3062. 11312290 10.1523/JNEUROSCI.21-09-03052.2001 PMC6762546

[97] Körber C, Werner M, Hoffmann J, et al. Stargazin interaction with alpha-amino-3-hydroxy-5-methyl-4-isoxazole propionate (AMPA) receptors is critically dependent on the amino acid at the narrow constriction of the ion channel. J Biol Chem. 2007 6 29;282(26):18758–18766. 17483093 10.1074/jbc.M611182200

[98] Osten P, Khatri L, Perez JL, et al. Mutagenesis reveals a role for ABP/GRIP binding to GluR2 in synaptic surface accumulation of the AMPA receptor. Neuron. 2000 8;27(2):313–325. 10985351 10.1016/s0896-6273(00)00039-8

[99] Fang L, Wu J, Zhang X, et al. Increased phosphorylation of the GluR1 subunit of spinal cord alpha-amino-3-hydroxy-5-methyl-4-isoxazole propionate receptor in rats following intradermal injection of capsaicin. Neuroscience. 2003;122(1):237–245. 14596864 10.1016/s0306-4522(03)00526-8

[100] Fang L, Wu J, Lin Q, et al. Protein kinases regulate the phosphorylation of the GluR1 subunit of AMPA receptors of spinal cord in rats following noxious stimulation. Brain Res Mol Brain Res. 2003 10 21;118(1–2):160–165. 14559367 10.1016/j.molbrainres.2003.08.002

[101] Miletic G, Hermes JL, Bosscher GL, et al. Protein kinase C gamma-mediated phosphorylation of GluA1 in the postsynaptic density of spinal dorsal horn neurons accompanies neuropathic pain, and dephosphorylation by calcineurin is associated with prolonged analgesia. Pain. 2015 12;156(12):2514–2520. 26270583 10.1097/j.pain.0000000000000323 PMC4653070

[102] Wang Y, Wu J, Guo R, et al. Surgical incision induces phosphorylation of AMPA receptor GluR1 subunits at Serine-831 sites and GluR1 trafficking in spinal cord dorsal horn via a protein kinase Cγ-dependent mechanism. Neuroscience. 2013 6 14;240:361–370. 23470774 10.1016/j.neuroscience.2013.02.051

[103] Boehm J, Kang MG, Johnson RC, et al. Synaptic incorporation of AMPA receptors during LTP is controlled by a PKC phosphorylation site on GluR1. Neuron. 2006 7 20;51(2):213–225. 16846856 10.1016/j.neuron.2006.06.013

[104] Peng HY, Chang CH, Tsai SJ, et al. Protein kinase A-dependent spinal α-amino-3-hydroxy-5-methyl-4-isoxazoleproprionate-receptor trafficking mediates capsaicin-induced colon-urethra cross-organ reflex sensitization. Anesthesiology. 2011 1;114(1):70–83. 21169799 10.1097/ALN.0b013e3181fe4204

[105] Jones TL, Sorkin LS, Activated PKA. PKC, but not CaMKIIalpha, are required for AMPA/Kainate-mediated pain behavior in the thermal stimulus model. Pain. 2005 10;117(3):259–270. 16150547 10.1016/j.pain.2005.06.003

[106] Fang L, Wu J, Lin Q, et al. Calcium-calmodulin-dependent protein kinase II contributes to spinal cord central sensitization. J Neurosci. 2002 5 15;22(10):4196–4204. 12019337 10.1523/JNEUROSCI.22-10-04196.2002 PMC6757653

[107] Jones TL, Lustig AC, Sorkin LS. Secondary hyperalgesia in the postoperative pain model is dependent on spinal calcium/calmodulin-dependent protein kinase II alpha activation. Anesth Analg. 2007 12;105(6):1650–1656. table of contents. 18042863 10.1213/01.ane.0000287644.00420.49

[108] Guire ES, Oh MC, Soderling TR, et al. Recruitment of calcium-permeable AMPA receptors during synaptic potentiation is regulated by CaM-kinase I. J Neurosci. 2008 6 4;28(23):6000–6009. 18524905 10.1523/JNEUROSCI.0384-08.2008 PMC2671029

[109] Barria A, Muller D, Derkach V, et al. Regulatory phosphorylation of AMPA-type glutamate receptors by CaM-KII during long-term potentiation. Science. 1997 6 27;276(5321):2042–2045. 9197267 10.1126/science.276.5321.2042

[110] Lee HK, Takamiya K, Han JS, et al. Phosphorylation of the AMPA receptor GluR1 subunit is required for synaptic plasticity and retention of spatial memory. Cell. 2003 3 7;112(5):631–643. 12628184 10.1016/s0092-8674(03)00122-3

[111] Esteban JA, Shi SH, Wilson C, et al. PKA phosphorylation of AMPA receptor subunits controls synaptic trafficking underlying plasticity. Nat Neurosci. 2003 2;6(2):136–143. 12536214 10.1038/nn997

[112] Kopach O, Krotov V, Belan P, et al. Inflammatory-induced changes in synaptic drive and postsynaptic AMPARs in lamina II dorsal horn neurons are cell-type specific. Pain. 2015 3;156(3):428–438. 25599231 10.1097/01.j.pain.0000460318.65734.00

[113] Kopach O, Medvediev V, Krotov V, et al. Opposite, bidirectional shifts in excitation and inhibition in specific types of dorsal horn interneurons are associated with spasticity and pain post-SCI. Sci Rep. 2017 7 19;7(1):5884. 28724992 10.1038/s41598-017-06049-7 PMC5517549

[114] Yasaka T, Tiong SY, Hughes DI, et al. Populations of inhibitory and excitatory interneurons in lamina II of the adult rat spinal dorsal horn revealed by a combined electrophysiological and anatomical approach. Pain. 2010 11;151(2):475–488. 20817353 10.1016/j.pain.2010.08.008 PMC3170912

[115] Lu Y. Modular organization of excitatory circuits between neurons of the spinal superficial dorsal horn (laminae I and II). J Neurosci. 2005 4 13;25(15):3900–3907. 15829642 10.1523/JNEUROSCI.0102-05.2005 PMC6724918

[116] Albuquerque C, Lee CJ, Jackson AC, et al. Subpopulations of GABAergic and non-GABAergic rat dorsal horn neurons express Ca2+-permeable AMPA receptors. Eur J Neurosci. 1999 8;11(8):2758–2766. 10457172 10.1046/j.1460-9568.1999.00691.x

[117] Kerr RC, Maxwell DJ, Todd AJ. GluR1 and GluR2/3 subunits of the AMPA-type glutamate receptor are associated with particular types of neurone in laminae I-III of the spinal dorsal horn of the rat. Eur J Neurosci. 1998 1;10(1):324–333. 9753141 10.1046/j.1460-9568.1998.00048.x

[118] Leitner J, Westerholz S, Heinke B, et al. Impaired excitatory drive to spinal GABAergic neurons of neuropathic mice. PLoS One. 2013;8(8):e73370. 24009748 10.1371/journal.pone.0073370 PMC3751881

[119] Coull JA, Boudreau D, Bachand K, et al. Trans-synaptic shift in anion gradient in spinal lamina I neurons as a mechanism of neuropathic pain. Nature. 2003 8 21;424(6951):938–942. 12931188 10.1038/nature01868

[120] Hildebrand ME, Xu J, Dedek A, et al. Potentiation of synaptic GluN2B NMDAR currents by fyn kinase is gated through BDNF-mediated disinhibition in spinal pain processing. Cell Rep. 2016 12 6;17(10):2753–2765. 27926876 10.1016/j.celrep.2016.11.024

[121] Moore KA, Kohno T, Karchewski LA, et al. Partial peripheral nerve injury promotes a selective loss of GABAergic inhibition in the superficial dorsal horn of the spinal cord. J Neurosci. 2002 8 1;22(15):6724–6731. 12151551 10.1523/JNEUROSCI.22-15-06724.2002 PMC6758148

[122] Dedek A, Xu J, Kandegedara CM, et al. Loss of STEP61 couples disinhibition to N-methyl-d-aspartate receptor potentiation in rodent and human spinal pain processing. Brain. 2019 6 1;142(6):1535–1546. 31135041 10.1093/brain/awz105 PMC6536915

[123] Inquimbert P, Moll M, Latremoliere A, et al. NMDA receptor activation underlies the loss of spinal dorsal horn neurons and the transition to persistent pain after peripheral nerve injury. Cell Rep. 2018 5 29;23(9):2678–2689. 29847798 10.1016/j.celrep.2018.04.107 PMC6276118

[124] Somers DL, Clemente FR. Dorsal horn synaptosomal content of aspartate, glutamate, glycine and GABA are differentially altered following chronic constriction injury to the rat sciatic nerve. Neurosci Lett. 2002 5 3;323(3):171–174. 11959412 10.1016/s0304-3940(02)00157-x

[125] Inquimbert P, Bartels K, Babaniyi OB, et al. Peripheral nerve injury produces a sustained shift in the balance between glutamate release and uptake in the dorsal horn of the spinal cord. Pain. 2012 12;153(12):2422–2431. 23021150 10.1016/j.pain.2012.08.011 PMC3540793

[126] NJ E, CA R, Fine A, et al. Optical quantal analysis reveals a presynaptic component of LTP at hippocampal Schaffer-associational synapses. Neuron. 2003 6 5;38(5):797–804. 12797963 10.1016/s0896-6273(03)00325-8

[127] Malgaroli A, AE T, Wendland B, et al. Presynaptic component of long-term potentiation visualized at individual hippocampal synapses. Science. 1995 6 16;268(5217):1624–1628. 7777862 10.1126/science.7777862

[128] Lozovaya NA, Kopanitsa MV, Boychuk YA, et al. Enhancement of glutamate release uncovers spillover-mediated transmission by N-methyl-D-aspartate receptors in the rat hippocampus. Neuroscience. 1999;91(4):1321–1330. 10391439 10.1016/s0306-4522(98)00638-1

[129] Kerchner GA, Nicoll RA. Silent synapses and the emergence of a postsynaptic mechanism for LTP. Nat Rev Neurosci. 2008 11;9(11):813–825. 18854855 10.1038/nrn2501 PMC2819160

[130] Kullmann DM, Asztely F. Extrasynaptic glutamate spillover in the hippocampus: evidence and implications. Trends Neurosci. 1998 1;21(1):8–14. 9464678 10.1016/s0166-2236(97)01150-8

[131] Twomey EC, Yelshanskaya MV, Vassilevski AA, et al. Mechanisms of channel block in. Neuron. 2018 9 5;99(5):956–968.e4. 30122377 10.1016/j.neuron.2018.07.027 PMC6181147

[132] Zaitsev AV, Kim KK, Fedorova IM, et al. Specific mechanism of use-dependent channel block of calcium-permeable AMPA receptors provides activity-dependent inhibition of glutamatergic neurotransmission. J Physiol. 2011 4 1;589(Pt 7):1587–1601. 21486838 10.1113/jphysiol.2011.204362 PMC3099017

[133] Christensen NR, De Luca M, Lever MB, et al. A high-affinity, bivalent PDZ domain inhibitor complexes PICK1 to alleviate neuropathic pain. EMBO Mol Med. 2020 6 8;12(6):e11248. 32352640 10.15252/emmm.201911248 PMC7278562

[134] Liu TY, Cheng Y, Qin XY, et al. Pharmacologically inhibiting GluR2 internalization alleviates neuropathic pain. Neurosci Bull. 2015 10;31(5):611–616. 26248656 10.1007/s12264-015-1556-2 PMC5563682

[135] Sullivan SJ, Farrant M, Cull-Candy SG. TARP γ-2 is required for inflammation-associated AMPA receptor plasticity within lamina ii of the spinal cord dorsal horn. J Neurosci. 2017 6 21;37(25):6007–6020. 28559374 10.1523/JNEUROSCI.0772-16.2017 PMC5481940

[136] Knopp KL, Simmons RMA, Guo W, et al. Modulation of TARP γ8-Containing AMPA Receptors as a Novel Therapeutic Approach for Chronic Pain. J Pharmacol Exp Ther. 2019 6;369(3):345–363. 30910921 10.1124/jpet.118.250126

